# CancelRx Case Study: Implications for Clinic and Community Pharmacy Work Systems

**DOI:** 10.21203/rs.3.rs-2859918/v1

**Published:** 2023-05-03

**Authors:** Taylor L. Watterson, Jamie A. Stone, Peter Kleinschmidt, Michelle A. Chui

**Affiliations:** University of Wisconsin School of Medicine and Public Health; University of Wisconsin-Madison School of Pharmacy; University of Wisconsin School of Medicine and Public Health; University of Wisconsin-Madison School of Pharmacy

**Keywords:** Health IT, Systems Approach, Case Study, Pharmacy

## Abstract

**Background:**

The medication prescribing, and de-prescribing process is complex with numerous actors, organizations, and health information technology (IT). CancelRx is a health IT that automatically communicates medication discontinuations from the clinic electronic health record to the community pharmacy’s dispensing platform, theoretically improving communication. CancelRx was implemented across a Midwest academic health system in October 2017.

**Objective:**

The goal of this study was to describe how both the clinic and community pharmacy work systems change and interact over time regarding medication discontinuations.

**Approach:**

Medical Assistants (n = 9), Community Pharmacists (n = 12), and Pharmacy Administrators (n =3), employed by the health system were interviewed across 3-time periods— 3-months prior to CancelRx implementation, 3-months after CancelRx implementation, and 9-months after CancelRx implementation. Interviews were audio recorded, transcribed, and analyzed via deductive content analysis.

**Key Results:**

CancelRx changed the medication discontinuation process at both clinics and community pharmacies. In the clinics, the workflows and medication discontinuation tasks changed over time while MA roles and clinic staff communication practices remained variable. In the pharmacy, CancelRx automated and streamlined how medication discontinuation messages were received and processed, but also increased workload for the pharmacists and introduced new errors.

**Conclusions:**

This study utilizes a systems approach to assess disparate systems within a patient network. Future studies may consider health IT implications for systems that are not in the same health system as well as assessing the role of implementation decisions on health IT use and dissemination.

## Background

Health care and health care delivery are complex due to the numerous actors, organizations, and systems involved in the medication prescribing process. From inception to the patient’s bedside, prescribing a medication requires connectivity between numerous systems: clinic professionals, pharmacy professionals, clinic and pharmacy health information technology (health IT), health insurance payers, patients, and their caregivers.

**Figure 1:**
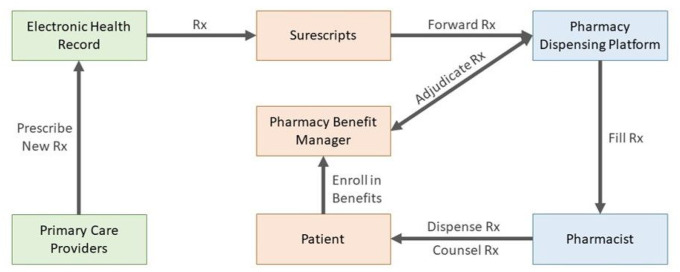
Simplified Medication Prescribing Process

As an example, when a patient attends their annual physical, they interact with clinic staff and their clinician or primary care provider (PCP). The PCP decides to prescribe a new medication. They document information into the clinic’s electronic health record (EHR) and input the prescription to be electronically sent to the patient’s pharmacy. The prescription “travels” through a third-party prescription vendor that acts as an intermediary between the EHR and the patient’s pharmacy dispensing platform. The patient’s pharmacy electronically receives the prescription where a pharmacy staff member inputs the information into the pharmacy dispensing platform and bills the patient’s insurance for the drug. The pharmacy staff packages the prescription which is then assessed by the pharmacist for accuracy and clinical appropriateness. Finally, the pharmacist dispenses the prescription to the patient, or their caregiver, and counsels them on appropriate use of the medication. The medication process is modeled in Fig. 1.

These multiple systems are important because they allow for specialization and expertise. However, when information is not shared or communicated effectively, there are risks to patient safety.

### Medication Discontinuation Process

Medication discontinuation refers to when a healthcare provider elects to change or stop a patient’s medication regimen. Reasons for medication discontinuation often include: completion of therapy or clinical improvement; medication related adverse effects, drug interactions, or allergies; or change in therapy for an alternative dose or product.(1)

When medication discontinuations are not communicated to pharmacies, patients’ medication records are inaccurate and makes pharmacies vulnerable to dispensing medications that should have been stopped.(2, 3) Despite clinic EHR discontinuation, a 2003 study found that 5% of medications supposed to be stopped were later dispensed to patients at the pharmacy.(4)

### CancelRx

A recent change to the medication discontinuation process, spanning clinic and pharmacy systems, is CancelRx. CancelRx is a health IT functionality that electronically deactivates the patient’s prescription when the clinic provider discontinues a medication at the clinic.(5, 6) Similar to new electronic prescriptions, CancelRx messages “travel” via the third-party platform, SureScripts, which links the clinic’s EHR to the pharmacy’s dispensing software. Because new electronic prescriptions are also sent via SureScripts, CancelRx messages can automatically match to active prescriptions in the pharmacy dispensing software, alert pharmacy staff, and automatically discontinue them—preventing future dispensing to patients.

CancelRx has been implemented in many health care organizations and community pharmacies and its use is recommended by the National Council on Prescription Drug Programs SCRIPT guidelines, incorporated into the Department of Health and Human Services 2015 Edition Health Information Technology Certification Criteria, and CMS 2017 Stage 3 Meaningful Use EHR Criteria.(5, 7, 8) A 2021 study reports an immediate, significant, and sustained increase in medication discontinuation between clinics and pharmacies after CancelRx implementation.(9)

One significant barrier to broader adoption of CancelRx is the concern about how it might impact workflow and workload in clinic and pharmacy work systems.(9, 10) CancelRx presents an opportunity to assess how novel health IT uniquely impacts and connects disparate systems and provide guidance for the design and implementation of future health IT.

#### Objective

The goal of this study was to describe how both the clinic and community pharmacy work systems change and interact over time regarding medication discontinuations.

## Methods

### Setting

This study was conducted within [Affiliation], the integrated health system of the [Affiliation]. [Affiliation] serves more than 600,000 patients each year with six hospitals and 80 outpatient sites. [Affiliation] also includes 15 outpatient community pharmacies which fill approximately 500,000 prescriptions annually. At the time of the study, [Affiliation] utilized Epic (Epic Systems Inc., Verona, WI) as the EHR vendor and EnterpriseRx (McKesson, San Francisco, CA) as the pharmacy dispensing software vendor.

[Affiliation] implemented CancelRx across the entire organization in October 2017 as part of a larger health IT upgrade. This study is specific to the [Affiliation] system as well their clinic and pharmacy health IT vendors.

### Data Collection

The research team received approval by the [Affiliation] Institutional Review Board prior to data collection. Data collection took place at 3 distinct time periods: at 3-months prior to CancelRx implementation, at 3-months after CancelRx implementation, and 9-months after CancelRx implementation.

The research team utilized [Affiliation] stakeholders to recruit 3 medical assistants (MAs) at each of 3 outpatient clinics, and 3 pharmacists at each of 3 outpatient pharmacies to participate in the study. At [Affiliation], MAs were the individuals primarily responsible for contacting the pharmacy when a medication was discontinued (pre-intervention). Pharmacists were the individuals primarily responsible for receiving or managing medication discontinuation messages (pre-intervention). The research team conducted semi-structured interviews to ascertain how medication discontinuation orders were received, prioritized, addressed, and communicated, as well as facilitators and barriers to communicating medication discontinuation messages to pharmacies or discontinuing the messages in the EnterpriseRx software. Additionally, interviews discussed the impact of medication discontinuation messages on workflow, workload, and their perceived outcomes.

At 9-months post CancelRx, pharmacy administrators were also interviewed to capture organization wide decisions and outcomes related to CancelRx implementation.

All interviews were audio recorded and transcribed via a professional agency.

### Data Analysis

The research team used QSR NVivo to analyze the semi-structured interview transcripts. TW and JS conducted qualitative deductive content analysis using the Systems Engineering Initiative for Patient Safety (SEIPS) model.(11–13) Within the SEIPS model, a system is comprised of five interconnected social and technical components (person, organization, technologies and tools, tasks, and [physical] environment) that impact how care is provided and the resulting patient outcomes.(11) The research team created codes based on the SEIPS components as well as sub-codes as identified in the literature. (11, 12) The research team also added inductive codes as needed. The completed codebook can be found in Appendix A.

The two coders (TW and JS) established consensus and common mental models. They then independently coded several transcripts to establish interrater reliability (Cohen’s Kappa calculated via QSR NVivo and determined sufficient > 0.80).(14–16) They independently coded the remaining transcripts, and returned to review transcript sections that required additional discussion. After coding was complete, the researchers independently reviewed the finding and then met to aggregate the key codes into overarching themes to address the study aims.

## Results

### The clinic system

A total of 9 medical assistants were interviewed. On average respondents were 38 years old (SE 3.11, median 36 years old) and had 14 years of experience working in a clinic setting (SE 2.97, median 13 years). All MA participants were female, white, and were not Hispanic or Latino.

The MA interviews yielded 2 key themes pertinent to how the clinic system changed over time: 1) workflow and tasks regarding medication discontinuations changed for clinic staff over time and 2) clinic staff roles, relationships, and communication patterns regarding medication discontinuations were variable and the variability persisted over time.

### Workflows and tasks regarding medication discontinuations changed for clinic staff over time

In general, there are several distinct workflows and accompanying tasks that occur when medications are discontinued at the clinic: first the medication is discontinued, second, the MA is made aware of the discontinuation, and third, the clinic communicates the medication discontinuation to the pharmacy. The tasks involved with these workflows changed over time, namely before and after CancelRx implementation.

### Pre-CancelRx

MAs received notification of a medication discontinuation task via a message sent through the EHR called an “in-basket message” in a folder titled “medication discontinuation.” Although all the MAs within a clinic received all the in-basket messages as part of a centralized queue, most MAs reported only opening messages belonging to their assigned providers and patients. The in-basket message informed the MA that a patient’s medication was discontinued and that they should contact the patient’s community pharmacy. The MAs indicated that this process could become time consuming, especially if they were placed on hold at the pharmacy or transferred between staff members. Within the in-basket message, MAs were able to document notes regarding the encounter, such as whether they called the pharmacy, who they spoke to at the pharmacy, etc.

Interviews with MAs indicated there was variability in how these in-basket messages were handled, including several who were unfamiliar with the medication discontinuation folder and unaware of the in-basket messages informing them to contact the pharmacy. Most MAs reported that they did not call the pharmacy on every in-basket message. Most MAs utilized professional judgement or clinical decision making to determine when to contact the pharmacy and when to mark the messages as “Done” without contacting the pharmacy.

It’s up to the provider if they discontinue it. If they do, it goes into a medication cancellation folder. And then it all depends if it’s a newer medication, and if it has refills, then we call the pharmacy and have them cancel the refills. Otherwise, if it’s a prescribed med that’s from two years ago, we just “done” it because there shouldn’t be refills left on it.

-Medical Assistant, Pre-CancelRx

### Post-CancelRx

CancelRx automatically communicated medication discontinuations between the clinic EHR and pharmacy dispensing platforms, potentially reducing the number instances where MAs were required to contact the pharmacy. However, medication discontinuation messages sent via CancelRx still appeared in the MA’s in-basket folder—informing them that the message had been communicated, as well as if and when the pharmacy discontinued the medication.

One MA reported that there were minimal differences between how the pre-CancelRx and CancelRx messages looked in the in-basket folder. Another MA stated that they knew which pharmacies were able to receive CancelRx messages and when they saw an in-basket message directing them to contact a CancelRx pharmacy they immediately “done [the messages] out.” Other MAs interviewed were not aware of the CancelRx functionality, even 3-months after implementation.

At 9-months after CancelRx implementation, most MAs were aware of the CancelRx functionality. Some MAs also shared how their trust (or lack thereof) in the CancelRx technology influenced their practice— they continued to call on all in-basket messages regardless of CancelRx status or they no longer called on CancelRx messages because they were confident the pharmacy received the medication discontinuation.

And some [messages] will say ‘cannot cancel’ and then list the reason. Or please call the pharmacy. So we call anyways. Then I call [the pharmacy] and sometimes what happens is that prescription is no longer there. They transferred it. If a patient transfers [a prescription] on their own, we don’t see that. And [the pharmacy] lets us know where it’s at, and then we can cancel it there.

-Medical Assistant, 9-Months Post-CancelRx

Clinic Staff Roles, Relationships and Communication Patterns Regarding Medication Discontinuations were Variable and the Variability Persisted Over Time

Within [Affiliation] clinics, MAs were partnered with providers to see patients, document encounters, and complete administrative tasks. MA roles and communication patterns, as they related to medication discontinuations, were variable based on these physician partnerships. This variability existed prior to CancelRx implementation and persisted after its implementation.

### Pre-CancelRx

Within the [Affiliation] organization, there was ambiguity as to whether or not MAs were permitted to discontinue medications from a patient’s profile. Some MAs stated they were not allowed to discontinue medications from a patient’s profile under any circumstances while others reported that they were allowed to discontinue patient reported medications such as over-the-counter products or historical medications from outside [Affiliation]. Other MAs indicated that they had agreements in place with their partnered provider that they were allowed to discontinue medications under certain conditions.

MAs utilized the EHR to communicate with their providers—updating the patient medication taking behavior or documenting findings within the encounter note. Some MAs also indicated that they would debrief with their providers in-person either before or after the provider saw the patient to review the patient’s medication list, discuss new, changed, or discontinued medications and if they need to contact the pharmacy. Providers would also use the EHR to communicate and share tasks with MAs and clinic staff members via the in-basket messaging—alerting MAs to contact the patient’s pharmacy when a medication was discontinued.

We’re not allowed to take it off the med list, so I’ll just click, ‘not taking’, and put it in my note and hope the doctor takes it off. […] Only if it’s over the counter. Then I’ll take it off the med list, but if it’s a prescribed medicine, we’re not supposed to touch it.

-Medical Assistant, Pre-CancelRx

### Post-CancelRx

After CancelRx implementation, variability still existed regarding whether MAs could remove medications from a patient’s EHR record. More MAs reported having agreements with their providers that allowed them to remove outdated or discontinued medications from the patient’s medication list.

MAs and providers still utilized the EHR functionality to communicate within the clinic when medications were changed or discontinued. MAs still received in-basket messages, even when CancelRx messages were sent and so the new functionality did not markedly change communication practices.

Well, I’m not supposed to [discontinue medications]. But the providers that I work with, and I have an agreement. Because we had noticed that a lot of times on the patient’s medication list if I marked not taking, it just sits there. So the patient will come in once a month for follow-ups, and I have to go over that in the med list. “Okay, so you’re still not taking such-and-such medication?” Because the provider just never takes it out. So the providers that I work with have given me the okay to go ahead and discontinue medications, as long as, it’s based on what the patient says.

-Medical Assistant 3-Months Post-CancelRx

#### The pharmacy system.

A total of 12 pharmacists and 3 pharmacy administrators (n = 15) were interviewed. The average age of the pharmacy staff members was 41.87 (SE 2.55, median 43 years old). On average, pharmacy staff participants had 14.73 years of experience working in a pharmacy setting (SE 2.28, median 13). Over half of the participants were female (n = 10, 67%), almost all were white (n = 14, 93% white; 1 Asian), and none of the participants were Hispanic or Latino.

A medication is successfully discontinued when it is cancelled in both the clinic EHR and pharmacy dispensing platforms.(9) The pharmacist interviews elucidated themes regarding 1) changes to pharmacy medication discontinuation workflow and 2) changes to patient safety concerns over time.

### Work ows and tasks regarding medication discontinuations changed for pharmacy staff over time

During the pre-CancelRx interviews, pharmacists described their process for receiving discontinuation messages that were sent from the clinic. First, they received the message (either via phone call, voicemail, fax, notes on prescriptions, or patient report), then they navigated to the patient profile in the pharmacy dispensing software. Pharmacists reported that most discontinuation messages did not include a reason for discontinuation and that, occasionally, they investigated the discontinuation message by reviewing the patient’s profile in the clinic EHR to read the provider’s notes. Finally, the pharmacists deactivated the prescription in the pharmacy dispensing software, cancelling future fills and refills of the medication, and removing it from the active medication list. The pharmacists sometimes documented the discontinuation via a note in the patient’s profile or informed other pharmacy staff members such as technicians or other pharmacists.

After CancelRx implementation, pharmacists reported that the main way they received discontinuation messages was electronically via CancelRx and that these CancelRx messages did not include a reason for why the prescription was cancelled. Pharmacists reported that the CancelRx functionality was able to automatically find and match discontinuation messages to linked prescriptions in the pharmacy’s dispensing profile and immediately discontinue the medication. Pharmacists were still required to review and attest to the CancelRx messages. However, once matched, they were unable to “un-discontinue” a CancelRx prescription.

Administration deemed reviewing and attesting to CancelRx messages was to be a “pharmacist only” task, as it required pharmacist clinical judgement and expertise.

### Medication Discontinuation Patient Safety Concerns Changed Over Time

Pharmacists reported problems and patient safety concerns related to the medication discontinuation process both prior to, and after CancelRx implementation. The problems tended to fluctuate from not receiving enough discontinuation messages pre-CancelRx to receiving too many unimportant discontinuation messages post-CancelRx and other technological challenges.

### Pre-CancelRx

Prior to CancelRx, pharmacists reported that medication discontinuation messages were generally not communicated reliably or consistently. This communication failure warranted medication safety concerns when patients were unsure of their medication regimens.

There’s certainly been an opportunity for potential for harm. So, there’s an atenolol shortage right now, so patients are getting switched from atenolol to other beta blockers. So, in this case it was metoprolol. The patient wasn’t aware that they were being switched off it. She kept calling in her atenolol. She had been sent the metoprolol, and then she was sent a supply of atenolol. So now she’s getting a double beta blocker, potentially.

-Pharmacist Pre-CancelRx

### Post-CancelRx

After CancelRx implementation, pharmacists commented on the increased volume of CancelRx messages, particularly on the increased number of messages that were merely “nuisances.” Pharmacists indicated messages for acute or completed therapies were often unnecessary and warranted them to move quickly through the queue of CancelRx messages.

Can I be embarrassing and say I don’t really even read them anymore? […] Well, it’s sad, but they’ve become more of a nuisance to me. I look to see how old the prescription is, what it’s for. It’s usually for a script that’s old or a non-maintenance med. .. or an antibiotic or something that, it doesn’t matter that it has been [discontinued]. The patient was on it short term. It should have been cleaned out of the profile ages ago, and it’s not appropriate to be sending us this message. […] It’s a very quick queue now. Where, in the beginning I was looking into the patient profile and going into HealthLink and trying to determine things, and now it’s just, oh, bogus, bogus, bogus. Get rid of it.

-Pharmacist, 9-Months Post-CancelRx

At 9-months post CancelRx, [Affiliation] pharmacy administrators described the cost-benefit tradeoff discussions with utilizing CancelRx, including discussions about turning the functionality off completely. The administrators reported that the benefits of CancelRx, including patient safety efforts, outweighed the cost (i.e., the noise of excess messages). An administrator also shared that if CancelRx was turned off, they feared providers at both [Affiliation] and other clinics would not know the functionality no longer worked, assume CancelRx messages were received at the pharmacy, and not communicate medication discontinuations via other means—thus posing more of a safety risk than pre-CancelRx practices.

We ran into the challenges with billing as well as the noise. And then also the concern about what’s our responsibility once we receive these messages? But ultimately, in the end, the pharmacists felt that there was still value in getting these messages and that they’ve had some good catches out of it. So, we stuck with it and continue to stick with it. And we felt strongly that the clinical piece trumped the operational piece. So it would have a lot easier for us to turn it off and pretend we don’t know, and it’s ne. But we knew that wasn’t the right thing to do, so we stuck with the clinical aspect even though it’s more busy work for IT. It’s more busy work for fiscal, and it might mean some bunch of false negatives we’re looking into. But for the few good ones, it was worth doing.

-Pharmacy Administrator 1 9-month post CancelRx

## Discussion

MA and pharmacist interviews identified changes that occurred in two disparate work systems—clinic and community pharmacies—when the same CancelRx functionality was implemented.

### Role of Technology in Linking Two Separate Systems

CancelRx had markedly different effects on the clinic and the pharmacy systems. For the MAs in the clinic setting, the medication discontinuation workload and tasks were slightly reduced after the implementation of CancelRx. Meanwhile, for the pharmacists in the pharmacy, the workload was markedly increased. The work was transferred from the clinic MA staff to the pharmacist.

While some MAs were unaware that CancelRx was implemented, pharmacists were calling CancelRx messages “nuisances” and administrators considered turning off the functionality completely. The case of CancelRx demonstrates how when two systems are linked, changes in one system may lead to unintended consequences in the other.

Administrators, executives, and researchers should consider other external and linked systems when implementing new technologies or services. As part of the patient journey, these unintended consequences may lead to patient safety vulnerabilities or negative outcomes.(17–20) Due to the large volume of CancelRx messages, pharmacists may have been de-sensitized to the non-useful messages (CancelRx messages for expired prescriptions or acute fills), a phenomenon known as alert fatigue.(21) Overwhelmed and fatigued, these pharmacists may have missed potentially crucial or more severe discontinuation messages that required complete attention. Identifying these vulnerabilities prior to turning on CancelRx may assist in making thoughtful implementation decisions to avoid alert fatigue risks. For example, setting system defaults to not automatically initiate CancelRx messages, only sending when prompted, and appropriately educating providers of the functionality. Similarly, CancelRx messaging may be set up to automatically send when specific reasons for discontinuation are selected, once again informing clinic staff of the need to accurately select reasons when discontinuing medications.

The case of CancelRx exemplifies the role for human factors engineering and other systems-based strategies such as proactive risk assessment when implementing novel technology, to consider not only unintended consequences within the system, but also in other externally linked systems that affect patient safety.(22) At Johns Hopkins, an interdisciplinary team conducted a proactive risk assessment as well as usability and pilot testing prior to CancelRx implementation.(10) They identified strategies to mitigate risks when implementing CancelRx including adding the reason for discontinuation to the CancelRx message and considering whether all prior prescriptions should be discontinued.

CancelRx exemplified the importance of considering unintended consequences of novel health IT in disparate systems within the same patient network. When assessing and mitigating unintended consequences of novel health IT, the tendency may be to focus on the identifying vulnerabilities in the functionality and usability of the technology itself. (23, 24) However, the policies and culture surrounding the technology use, are also crucial to consider.

CancelRx implementation both influenced and was influenced by [Affiliation] social systems. In the clinic, CancelRx was used differently depending on the role of the staff member. Providers, and sometimes MAs when permitted, were sending the CancelRx messages when discontinuing medications from the patient’s EHR record. MAs were responsible for following up or communicating medication discontinuation messages in the event of CancelRx failure. MAs reported instances when providers would not discontinue medications from the patient’s EHR profile. Some cited frustrations while others detailed agreements with their partnered providers to remove discontinued medications from the patient’s record. Assessing the appropriateness of [Affiliation]’s decision and provider practices regarding MA discontinuation of medications is beyond the scope of this study, however; CancelRx messages are not sent to the pharmacy if they are not discontinued from the EHR medication list.

### Limitations

The study captured data from clinics and community pharmacies located within one organization; the community pharmacies also had access to the clinic EHR data which is not common for all community pharmacies.

## Conclusion

CancelRx was implemented across [Affiliation] in October 2017 and changed the medication discontinuation process at both clinics and community pharmacies. In the clinics, the workflows and medication discontinuation tasks changed over time while MA roles and clinic staff communication practices remained variable. In the pharmacy, CancelRx automated and streamlined how medication discontinuation messages were received and processed, but also increased workload for the pharmacists and introduced new errors.

This study utilizes a systems approach to assess disparate systems within a patient network. Future studies may consider health IT implications for systems that are not in the same health system as well as assessing the role of implementation decisions on health IT use and dissemination.

## Data Availability

The data underlying this article cannot be shared publicly for the privacy of individuals that participated in the study. Deidentified will be shared on reasonable request to the corresponding author.
